# Epidemiology of human West Nile virus infections in the European Union and European Union enlargement countries, 2010 to 2018

**DOI:** 10.2807/1560-7917.ES.2021.26.19.2001095

**Published:** 2021-05-13

**Authors:** Johanna J Young, Joana M Haussig, Stephan W Aberle, Danai Pervanidou, Flavia Riccardo, Nebojša Sekulić, Tamás Bakonyi, Céline M Gossner

**Affiliations:** 1 European Centre for Disease Prevention and Control (ECDC), Solna, Sweden; 2 These authors contributed equally to this article and share first authorship; 3 Center for Virology, Medical University of Vienna, Vienna, Austria; 4 Hellenic National Public Health Organization, Athens, Greece; 5 Istituto Superiore di Sanità, Rome, Italy; 6 Institute for Public Health of Montenegro, Podgorica, Montenegro

**Keywords:** epidemiology, Europe, surveillance, vector-borne infections, West Nile fever, West Nile virus

## Abstract

**Background:**

West Nile virus (WNV) circulates in an enzootic cycle involving mosquitoes and birds; humans are accidental hosts.

**Aim:**

We analysed human WNV infections reported between 2010 and 2018 to the European Centre for Disease Prevention and Control to better understand WNV epidemiology.

**Methods:**

We describe probable and confirmed autochthonous human cases of WNV infection reported by European Union (EU) and EU enlargement countries. Cases with unknown clinical manifestation or with unknown place of infection at NUTS 3 or GAUL 1 level were excluded from analysis.

**Results:**

From southern, eastern and western Europe, 3,849 WNV human infections and 379 deaths were reported. Most cases occurred between June and October. Two large outbreaks occurred, in 2010 (n = 391) and in 2018 (n = 1,993). The outbreak in 2018 was larger than in all previous years and the first cases were reported unusually early. The number of newly affected areas (n = 45) was higher in 2018 than in previous years suggesting wider spread of WNV.

**Conclusion:**

Real-time surveillance of WNV infections is key to ensuring that clinicians and public health authorities receive early warning about the occurrence of cases and potential unusual seasonal patterns. Human cases may appear shortly after first detection of animal cases. Therefore, public health authorities should develop preparedness plans before the occurrence of human or animal WNV infections.

## Background

West Nile virus (WNV) infection is a zoonosis endemic in many parts of Europe, apart from northern Europe. The virus is primarily transmitted through the bites of infected mosquitoes, mainly of the *Culex* genus, but occasionally also through transfusion/transplantation of substances of human origin (SoHO) (i.e. blood, organs or cells), percutaneous exposure or inhalation in laboratories, or transplacental passage from mother to fetus [1]. Mosquitoes serve as vectors and birds are the main amplifying hosts [1]. Humans and other mammals, such as equids, are dead-end hosts [2]. In humans, the incubation period is usually 2–6 days, although incubation periods of up to 21 days have been reported in immunocompromised people [1]. Most humans infected with WNV remain asymptomatic, ca 20% develop influenza-like symptoms and less than 1% develop severe symptoms such as encephalitis, meningoencephalitis or meningitis [3]. Elderly and immunocompromised individuals are at higher risk of developing severe symptoms [4].

WNV lineages 1 and 2 are associated with human disease, with differences in virulence; however no clear linkage between virulence and lineage classification exists [1]. In Europe, WNV infections in humans were first detected by serological studies in Albania in 1958 [5,6]. A WNV strain of genetic lineage 1 was isolated for the first time in humans and mosquitoes in 1963 in the Rhône delta, France; it has since then caused sporadic cases and occasional outbreaks in animals and humans [6]. Lineage 2 was first detected in Hungary in 2004 and subsequently spread across central Europe and the eastern Mediterranean region [7,8] causing major outbreaks (e.g. Greece 2010, Serbia 2012 and several European countries 2018) [9-13]. The exact origin of the strain has not been identified. The nucleotide sequences had the highest similarity to WNV isolates from sub-Saharan Africa from the 1990s [10,12]. Therefore, it is hypothesised that the strain was introduced from Africa, most probably by migratory birds.

WNV infections have been notifiable at the European Union (EU) level since 2008 but only became notifiable in some EU countries at a later stage [14,15]. The EU countries report human cases to the European Centre for Disease Prevention and Control (ECDC) which, in turn, produces annual epidemiological summaries and, since 2011, weekly surveillance updates. Within the framework of the EU enlargement cooperation, EU enlargement countries also report human infections to ECDC. Reporting human WNV infections has been mandatory in Montenegro since 2012 and was introduced in Serbia in 2012 [16]. The main objective of timely WNV surveillance at the EU level is to provide early warning to public health professionals about areas with human WNV infections and thereby prevent human-to-human transmission via donation of contaminated SoHO. The EU blood safety directive obliges blood establishments to defer donors for 28 days after leaving an area where human cases were detected unless an individual donation nucleic acid test is negative [17].

We describe the epidemiology of WNV infections in EU and EU enlargement countries between 2010 and 2018 and raise hypotheses explaining the intensity of the transmission, the geographical spread of the virus and the seasonality of virus circulation. Finally, we highlight challenges and opportunities of strengthening WNV surveillance in Europe.

## Methods

Following the EU case definition (Commission Decision 2008/426/EC) [18], we included probable and confirmed autochthonous human cases of WNV infection reported to the ECDC between 2010 and 2018 by EU and EU enlargement countries.

### Operational definitions

Cases of WNV infection included cases with WNV neuroinvasive disease (WNND), cases with clinical signs or symptoms but without neurological manifestations (West Nile fever (WNF)) and asymptomatic cases. Cases with an unknown clinical manifestation were excluded. Autochthonous cases were individuals exposed in the reporting country during the incubation period of the infection. Asymptomatic cases were captured through blood donation screening. Affected areas were mapped at nomenclature of territorial units for statistics (NUTS) 3 level, or alternatively global administrative unit layers (GAUL) 1 level and were areas where (at least one) human autochthonous WNV infection occurred in a given year [19,20]. Newly affected areas were areas that were affected for the first time after 2010. Cases with an unknown place of infection at NUTS 3 or GAUL 1 level were excluded from all analyses.

### Data analysis

The following variables were analysed: demographical information (e.g. age, sex), case classification (confirmed or probable), clinical manifestation (WNND, WNF or asymptomatic), date of disease onset, date of diagnosis, date of hospitalisation, date of notification, reporting country, place of infection, importation status (autochthonous or travel-related) and outcome (survival or death).

The case fatality (CF) of cases with WNND was calculated by dividing the number of deaths by the number of WNND cases in a given year. The trend analysis was based on month of disease onset. When month of onset was missing, we used the month of diagnosis and ultimately the month of notification. Statistical analyses were conducted using STATA/IC version 13.0 (Stata Corp., College Station, United States). Maps were produced using the ECDC Map maker (EMMa) (ECDC, Solna, Sweden) [21].

## Results

### Epidemiological summary

Between 2010 and 2018, 13 EU countries (Austria, Bulgaria, Croatia, Cyprus, Czechia, France, Greece, Hungary, Italy, Portugal, Romania, Slovenia and Spain) and five EU enlargement countries (Albania, Montenegro, Serbia, Turkey and Kosovo*) reported 3,849 cases of human WNV infections with known clinical manifestation and place of infection (Table 1). Among these, 2,804 (73%) were WNND cases, 906 (24%) were WNF cases and 139 (4%) were asymptomatic (Table 2). In addition, 132 cases were reported that were excluded from the study because of missing information on exact place of infection and clinical manifestation. Greece (n = 978; 25%) and Italy (n = 971; 25%) reported the highest numbers of WNV infections with known clinical manifestation and place of infection, followed by Serbia (n = 613; 16%). Confirmed cases accounted for 78% of the cases (ranging from 50% in 2010 to 89% in 2016) and the proportion of confirmed cases increased over time.

**Table 1:** Autochthonous cases of West Nile virus infection with known clinical manifestations and place of infection reported at NUTS 3 or GAUL 1 level: case number, fatal cases, case fatality by country and year, EU and EU enlargement countries, 2010–2018 (n = 3,849)

		EU countries																										EU enlargement countries										Total	
		Austria		Bulgaria		Croatia		Cyprus		Czechia		France		Greece		Hungary		Italy		Portugal		Romania		Slovenia		Spain		Albania		Montenegro		Serbia		Turkey		Kosovo^a^			
		n	d	n	d	n	d	n	d	n	d	n	d	n	d	n	d	n	d	n	d	n	d	n	d	n	d	n	d	n	d	n	d	n	d	n	d	n	d
2010	Total	1	0	NR		NR		0	0	0	0	0	0	262	35	18	1	4	0	NR		57	5	0	0	2	0	NR		NR		NR		47	10	NR		391	51
	WNND	1	0	NR		NR		0	0	0	0	0	0	197	33	16	1	3	0	NR		57	5	0	0	2	0	NR		NR		NR		40	10	NR		316	49
	CF WNND	0%		NR		NA		NA		NA		NA		17%		6.3%		0%		NA		8.8%		NA		0%		NA		NA		NA		25%		NA		16%	
2011	Total	0	0	NR		NR		0	0	0	0	0	0	100	9	4	0	18	3	NR		11	1	0	0	0	0	15	0	NR		NR		1	0	NR		149	13
	WNND	0	0	NR		NR		0	0	0	0	0	0	74	9	4	0	14	3	NR		11	1	0	0	0	0	7	0	NR		NR		1	0	NR		111	13
	CF WNND	NA		NR		NA		NA		NA		NA		12%		0%		21%		NA		9.1%		NA		NA		0%		NA		NA		0%		NA		12%	
2012	Total	0	0	0	0	6	0	0	0	0	0	0	0	157	17	17	0	45	0	NR		15	1	0	0	0	0	0	0	1	0	0	0	NR		0	0	241	18
	WNND	0	0	0	0	6	0	0	0	0	0	0	0	106	17	8	0	28	0	NR		15	1	0	0	0	0	0	0	1	0	0	0	NR		0	0	164	18
	CF WNND	NA		NA		0%		NA		NA		NA		16%		0%		0%		NA		6.7%		NA		NA		NA		0%		NA		NA		NA		11%	
2013	Total	0	0	0	0	20	0	0	0	0	0	0	0	85	11	35	1	80	1	NR		24	0	1	0	0	0	0	0	3	0	0	0	NR		0	0	248	13
	WNND	0	0	0	0	20	0	0	0	0	0	0	0	50	10	32	1	44	1	NR		24	0	1	0	0	0	0	0	1	0	0	0	NR		0	0	172	12
	CF WNND	NA		NA		0%		NA		NA		NA		20%		3%		2.3%		NA		0%		0%		NA		NA		0%		NA		NA		NA		7.0%	
2014	Total	2	0	0	0	1	0	0	0	0	0	0	0	15	6	10	0	24	0	NR		23	1	0	0	0	0	0	0	0	0	77	8	NR		0	0	152	15
	WNND	1	0	0	0	1	0	0	0	0	0	0	0	14	6	6	0	21	0	NR		23	1	0	0	0	0	0	0	0	0	59	8	NR		0	0	125	15
	CF WNND	0%		NA		0%		NA		NA		NA		43%		0%		0%		NA		4.3%		NA		NA		NA		NA		14%		NA		NA		12.0%	
2015	Total	6	0	2	1	1	0	0	0	0	0	1	0	0	0	18	0	61	0	1	0	32	1	0	0	0	0	0	0	0	0	28	3	NR		0	0	150	5
	WNND	1	0	0	0	1	0	0	0	0	0	0	0	0	0	16	0	38	0	1	0	32	1	0	0	0	0	0	0	0	0	28	3	NR		0	0	117	4
	CF WNND	0%		NA		0%		NA		NA		NA		NA		0%		0%		0%		3.1%		NA		NA		NA		NA		11%		NA		NA		3.4%	
2016	Total	5	0	1	0	2	0	1	0	0	0	0	0	0	0	42	5	76	2	0	0	93	19	0	0	3	0	0	0	0	0	44	2	1	1	0	0	268	29
	WNND	1	0	0	0	2	0	1	0	0	0	0	0	0	0	33	5	39	2	0	0	93	19	0	0	2	0	0	0	0	0	41	2	1	1	0	0	213	29
	CF WNND	0%		NA		0%		0%		NA		NA		NA		15%		5%		NA		20%		NA		0%		NA		NA		4.9%		100%		NA		14%	
2017	Total	6	0	1	0	5	1	0	0	0	0	2	0	48	5	20	4	53	1	0	0	66	14	0	0	0	0	0	0	0	0	49	2	7	1	0	0	257	28
	WNND	2	0	1	0	5	1	0	0	0	0	0	0	28	5	17	4	26	1	0	0	66	14	0	0	0	0	0	0	0	0	48	2	4	1	0	0	197	28
	CF WNND	0%		0%		20%		NA		NA		NA		18%		24%		3.8%		NA		21%		NA		NA		NA		NA		4.2%		25%		NA		14%	
2018	Total	19	0	15	3	57	4	1	0	3	1	27	0	311	51	213	14	610	49	0	0	277	43	4	0	0	0	1	1	0	0	415	35	26	3	14	3	1,993	207
	WNND	4	0	13	3	46	4	1	0	3	1	7	0	240	48	152	14	243	49	0	0	277	43	4	0	0	0	1	1	0	0	368	33	18	3	12	3	1,389	202
	CF WNND	0%		23%		8.7%		0%		33%		0%		20%		9.2%		20%		NA		16%		0%		NA		100%		NA		9.0%		17%		25%		15%	
Total	Total	39	0	19	4	92	5	2	0	3	1	30	0	978	134	377	25	971	56	1	0	598	85	5	0	5	0	16	1	4	0	613	50	82	15	14	3	3,849	379
	WNND	10	0	14	3	81	5	2	0	3	1	7	0	709	128	284	25	456	56	1	0	598	85	5	0	4	0	8	1	2	0	544	48	64	15	12	3	2,804	370
	CF WNND	0%		21%		6.2%		0%		33%		0%		18%		8.8%		12%		0%		14%		0%		0%		13%		0%		8.8%		23%		25%		13%	

CF: case fatality; d: deaths; EU: European Union; n: number; GAUL: global administrative unit layers; NA: not applicable; NR: not reported; NUTS: nomenclature of territorial units for statistics; WNND: West Nile neuroinvasive disease.

^a^ This designation is without prejudice to positions on status, and is in line with United Nations Security Council Resolution 1244/99 and the International Court of Justice Opinion on the Kosovo declaration of independence.

**Table 2:** Main characteristics of cases of West Nile virus infection, EU and EU enlargement countries, 2010–2018 (n = 3,849)

	2010	2011	2012	2013	2014	2015	2016	2017	2018	2010–2018
Demographics										
Median age in years (IQR)	70 (53–77)	70 (50–77)	67 (54–76)	66 (51–78)	65 (53–74)	63 (49–74)	65 (53–75)	64 (51–76)	66 (52–76)	66 (52–76)
Female: age (IQR)	71 (58–78)	70 (48–77)	68 (53–77)	64 (49–79)	66 (53–75)	61 (45–69)	69 (54–77)	64 (51–76)	66 (50–77)	66 (51–77)
Male: age (IQR)	67 (50–76)	70 (52–75)	66 (54–75)	67 (53–77)	64 (52–74)	63 (51–76)	64 (52–74)	64 (50–77)	66 (54–76)	66 (53–76)
Male:female ratio	1.3	1.7	1.3	1.5	1.8	2.7	1.4	1.6	1.5	1.5
Clinical manifestation										
% WNND	81	74	68	69	82	78	79	77	70	73
% WNF	19	25	32	30	18	12	12	16	26	24
% Asymptomatic cases	0.0	0.67	0.0	0.40	0.0	10.0	8.6	7.0	4.1	3.6
Classification										
% Confirmed cases	50	66	52	73	81	86	89	77	86	78

EU: European Union; IQR: interquartile range; WNND: West Nile neuroinvasive disease.

The yearly number of reported WNV infections fluctuated, with peaks observed in 2010 (n = 391) and 2018 (n = 1,993) (Table 1). In 2010, Greece (n = 262; 67%) and Romania (n = 57; 15%) reported the highest number of cases. In 2018, Italy (n = 610; 31%), Serbia (n = 415; 21%) and Greece (n = 311; 16%) reported the highest number of cases. For the majority of the countries, 2018 was the year when the highest number of cases was reported. Between 2010 and 2018, 2,804 WNND cases were reported. Greece (n = 709; 25%), Romania (n = 598; 21%) and Serbia (n = 544; 19%) reported the majority of the WNND cases. The annual proportion of WNND cases ranged from 68% in 2018 to 82% in 2014. The proportion of cases with WNF ranged from 12% in 2015 and 2016 to 32% in 2012. Asymptomatic cases were reported between 2011 and 2018 and ranged between 0.67% in 2011 and 10% in 2015.

Between 2010 and 2018, 379 deaths among cases of WNV infection were reported (Table 1). The CF among WNND cases ranged from 3.4% in 2015 to 16% in 2010. The highest total CF among WNND cases were observed in Czechia (1/3), followed by Kosovo* (3/12) and Turkey (15/64), although the number of cases was very low limiting the interpretation of this CF.

### Demographic characteristics

The overall male:female ratio (M:F ratio) was 1.5:1, ranging from 1.3 in 2010 and 2012 to 2.7 in 2015. The M:F ratio among WNND cases was 1.6:1. The median age of cases of WNV infection was 66 years (interquartile range (IQR): 52–76) (Table 2), while the median age of cases with WNND was 69 years (IQR: 57–77). The median age of female and male cases of WNV infection was comparable. Between 2010 and 2018, the overall median age was 66 years for both female and male cases (Table 2).

### Trend and seasonality

**Figure 1:**
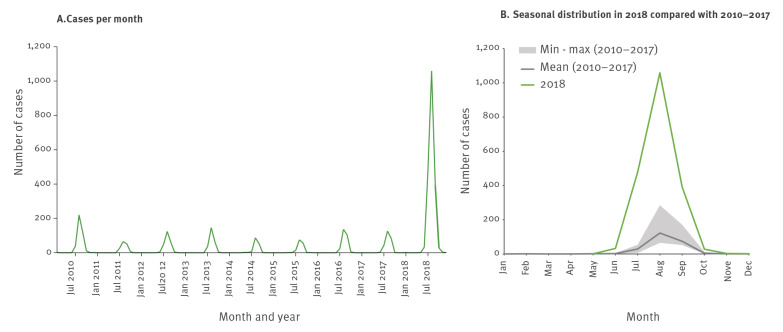
Reported cases of West Nile virus infection by month and year of disease onset^a^ and seasonal distribution, EU and EU enlargement countries, 2010–2018 (n = 3,849)

The month of disease onset was available for 96% of the cases. Alternate dates such as month of diagnosis and month of notification were used for 3.6% and 0.026% of the cases, respectively. WNV transmission followed a seasonal pattern: most infections occurred from early summer to early autumn, with a clear peak in August (Figure 1). In 2018, the transmission season was longer than in previous years, starting in week 22, about 2 weeks earlier than usual and lasting until week 49, around 9 weeks longer than usual.

EU: European Union.

^a ^Where month of disease onset was missing, we used month of diagnosis or month of notification (n = 138).

### Geographical distribution

The number of affected countries increased from seven countries in 2010 to 15 in 2018. Generally, when the number of reported infections increased, the number of affected areas increased accordingly. On average, 57 NUTS 3 areas were affected every year between 2010 and 2017 (range: 41–72), whereas in 2018, 169 areas were affected (Table 3). 

**Table 3:** Affected areas and newly affected areas (NUTS 3 and GAUL 1 levels) with West Nile virus infections, by country, EU and EU enlargement countries, 2010–2018 (n = 689)

		2010	2011		2012		2013		2014		2015		2016		2017		2018		2010–2018
Country	NUTS 3/ GAUL 1 areas in the country	Affected areas	Affected areas	Newly affected areas	Affected areas	Newly affected areas	Affected areas	Newly affected areas	Affected areas	Newly affected areas	Affected areas	Newly affected areas	Affected areas	Newly affected areas	Affected areas	Newly affected areas	Affected areas	Newly affected areas	% affected areas in the country
EU																			
Austria	35	1	0	0	0	0	0	0	2	1	2	1	2	0	3	1	5	2	17%
Bulgaria	28	0	0	0	0	0	0	0	0	0	2	2	1	0	1	1	6	5	29%
Croatia	21	0	0	0	3	3	3	3	1	0	1	0	1	0	4	1	11	5	57%
Cyprus	1	0	0	0	0	0	0	0	0	0	0	0	1	1	0	0	1	0	100%
Czechia	14	0	0	0	0	0	0	0	0	0	0	0	0	0	0	0	1	1	7.1%
France	102	0	0	0	0	0	0	0	0	0	1	1	0	0	1	1	5	4	5.9%
Greece	52	11	19	12	19	7	12	1	4	1	0	0	0	0	5	3	23	2	71%
Hungary	20	11	4	1	13	5	12	1	6	0	10	0	14	1	9	0	20	1	100%
Italy	110	3	7	6	9	4	16	12	11	2	15	4	16	3	18	2	33	6	38%
Portugal	25	0	0	0	0	0	0	0	0	0	1	1	0	0	0	0	0	0	4%
Romania	42	19	5	1	6	3	11	1	13	2	14	3	22	3	20	1	34	4	88%
Slovenia	12	0	0	0	0	0	1	1	0	0	0	0	0	0	0	0	4	4	42%
Spain	59	1	0	0	0	0	0	0	0	0	0	0	1	1	0	0	0	0	3.4%
EU enlargement																			
Albania	36	0	5	4	1	0	1	0	1	0	1	0	1	0	1	0	2	1	17%
Montenegro	21	0	0	0	1	1	3	2	0	0	0	0	0	0	0	0	0	0	14%
Serbia	25	0	0	0	0	0	0	0	9	9	7	2	7	1	6	2	17	6	80%
Turkey	81	15	1	0	0	0	0	0	0	0	0	0	1	0	5	4	6	3	27%
Kosovo^a^	5	0	0	0	0	0	0	0	0	0	0	0	0	0	0	0	1	1	20%
**TOTAL**	**689**	**61**	**41**	**24**	**52**	**23**	**59**	**21**	**47**	**15**	**54**	**14**	**67**	**10**	**73**	**16**	**169**	**45**	**34%**

GAUL: global administrative unit layers; EU: European Union; NUTS: nomenclature of territorial units for statistics.

^a^ This designation is without prejudice to positions on status, and is in line with United Nations Security Council Resolution 1244/99 and the International Court of Justice Opinion on the Kosovo declaration of independence.

**Figure 2:**
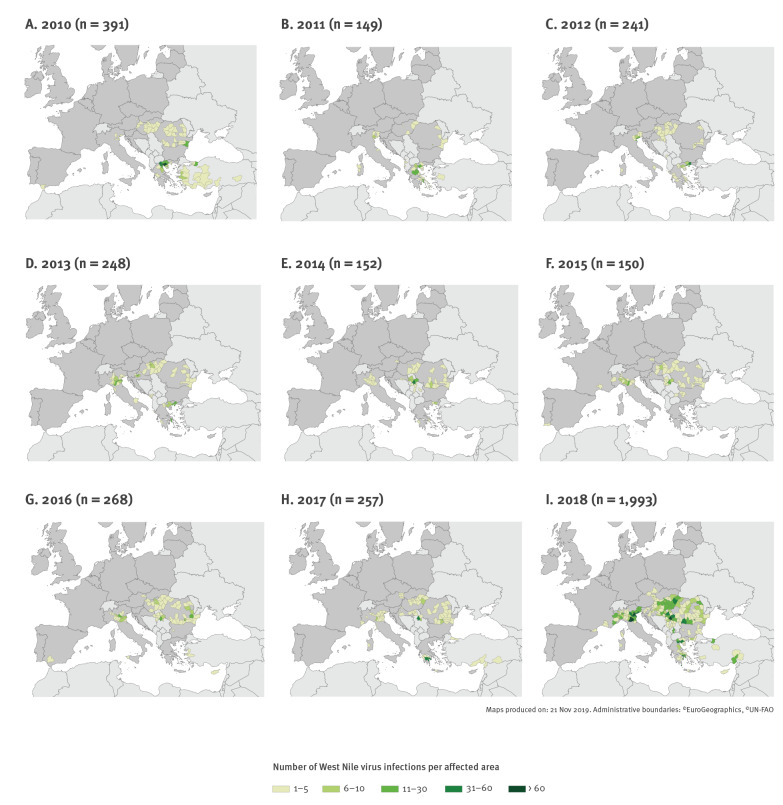
Geographical distribution of cases of West Nile virus infection by affected areas (NUTS 3 level or GAUL 1) and year, EU and EU enlargement countries, 2010–2018 (n = 3,849)

GAUL: global administrative unit layers; EU: European Union; NUTS: nomenclature of territorial units for statistics.

Over the study period, some affected countries detected a wide spread of WNV infections across the country. Especially in 2018 when WNV infections were more widely distributed than in previous years, cases were reported from the whole of Hungary and large parts of Romania, Serbia and Greece (Figure 2 and Table 3). However, other countries reported only a limited spread until the time of writing of this report, such as Spain, Portugal and France. Every year, new areas were affected (Table 3). Between 2011 and 2017, there were on average 18 (range: 10–24) newly affected areas annually. In comparison, 45 areas were newly affected in 2018.

## Discussion

Between 2010 and 2018, cases of WNV infection were reported every year with peaks in the number of cases in 2010 and 2018. This pattern resembles the WNV pattern in North America, where infections have been reported every year since it first emerged in 1999 and major outbreaks occur in certain years [22,23]. In 2018, Europe faced its largest WNV outbreak, with more cases reported in 2018 than the cumulative number of cases reported between 2010 and 2017.

The intensity of WNV transmission in a given year is determined by the abundance of competent mosquitoes and the prevalence of infection in them [24]. Higher temperatures can shorten the extrinsic incubation period of the virus in the vector [25], increase the vector population’s growth rate, accelerate the evolution rate of the virus and increase viral transmission to birds [26]. Increased precipitation positively correlates with disease outbreaks because of higher mosquito abundance, while drought may intensify bird–mosquito interaction around remaining water sources [25]. Weather conditions can also affect the reproduction patterns of the avian hosts [27-29]. For example, the date when the first eggs are laid can influence the number of nestlings in the beginning of the WNV transmission season and the number of broods within a season [27-29].

Increased intensity of virus circulation could be related to higher virulence of the circulating strains, leading to more severe disease and consequently more symptomatic infections. For example in Romania, in 2016 and 2017, an outbreak of WNND with high CF in humans was reported [30,31]. It followed the introduction of a new lineage 2 strain in the mosquito population in 2015 and its spread during the 2016 transmission season [30]. The circulating strain in 2018, however, was genetically closely related to the strain circulating during previous years and, to our knowledge, had no markers of higher virulence [32-37].

Background immunity against flaviviruses such as WNV influences the intensity of outbreaks in an endemic area [38]. In populations with background immunity, the number of symptomatic humans and equids is lower [39] and the reduced susceptible human population has been posited in one study as one of the likely factors behind the absence of reported infections in Greece between 2015 and 2016 [40]. Birds recovering from a WNV infection usually develop long-term immunity [41,42]. Reduced herd immunity in birds (i.e. after an increase in immunologically naïve offspring) can increase the risk of more intense virus circulation [43]. For example, the first occurrence of WNV infections in North America in 1999 lead to a major WNV outbreak with more than 20,000 human cases. Birds in this area had no previous exposure to WNV before this outbreak and the lack of acquired and evolved immunity may have increased the intensity of the epidemic [44]. Furthermore, the enzootic cycle of WNV might be influenced by immunological cross-reactivity to other flaviviruses in the Japanese encephalitis virus serocomplex, such as Usutu virus. Co-circulation of West Nile and Usutu viruses in the same ecosystem has been described without evidence of cross protection or antibody-dependant enhancement [45], and the avian host spectrum of the two viruses might not be the same.

The total number of affected areas and the number of newly affected areas in 2018 was higher than in all previous years, suggesting an increased spread of WNV. Furthermore, in 2018, human cases of WNV infection were reported from countries such as Czechia and Slovenia that had not previously reported human WNV infections. Often newly affected areas border on areas already affected in previous years, suggesting that the virus spreads from affected areas to non-affected neighbouring areas. The weighted average of the number of affected areas in neighbouring areas affected in previous years has been described as a risk factor for WNV circulation [46].

In 2018, WNV was detected for the first time in birds and equids in five federal states in Germany, and locally acquired human infections were reported in 2019 [47,48]. This highlights that once the enzootic cycle of the virus is established, infections among accidental hosts (i.e. equids and humans) can be expected.

We suggest that the spread of WNV in Europe is largely due to local movements of long-range migratory and resident birds, while migratory birds returning from their overwintering places in Africa or other regions probably played a minimal role in recent years as the virus is already endemic in Europe [49]. In 2010 in Romania, newly affected areas were known to be roosting places on bird migration pathways [50]. Overwintering of infected mosquitoes has an important role in the maintenance of WNV [51].

Changing weather conditions may affect migration patterns, and therefore the spread of the virus within Europe [25,52]. However, environmental and ecological conditions in Europe today are permissive for the overwintering and establishment of WNV strains [51] and the disease is considered endemic in the southern part of Europe. Genetic studies indicate that WNV infections after 2004 in central and southern Europe were predominantly caused by the descendants of the lineage 2 strain that emerged in 2004 and became endemic in the region [10,11,40,48,51,53,54].

The role of a bird species in the enzootic cycle of the virus depends on the number of birds and attractiveness to mosquitoes as well as their physiological capacity for transmitting the infection to mosquito vectors [55]. The primary avian host species that maintain an enzootic cycle in Europe remain unknown [26]. Testing the serological status of captive and wild birds could be a useful indicator for the estimation of host susceptibility to WNV infections and more research is needed to identify the potential capacity of different bird species to contribute to WNV circulation.

Generally, the transmission season in Europe lasts from June to October, with a peak in August [56]. In 2018, Greece, Hungary, Italy, Romania and Serbia observed a high number of cases very early in the transmission season [13,57]. Furthermore, infections occurred until the end of November, which marked an extended transmission season.

The timing of WNV outbreaks may be influenced by the arrival of migratory birds in locations close to or along migration routes during spring migration and by the abundance of amplification hosts among local birds. These could within 2–3 months infect large portions of the vector population and subsequently pass the virus to humans by June to September in the same year [53]. In recent years, because of an early rise of the mean spring temperatures, several bird species have been migrating to their breeding grounds earlier than previously observed [25]. 

The fact that WNV can overwinter in adult *Culex* mosquitoes [58] could also explain an earlier start of the transmission season, as vertically infected diapausing mosquitoes that emerged at the end of the breeding season can initiate earlier transmission in the following spring. Because many WNV infections remain asymptomatic, only a fraction is captured by surveillance. Considering that WNND reflects less than 1% of the total WNV infections [1], it can be estimated that the actual number of infections between 2010 and 2018 was at least 280,400.

The way countries conduct surveillance varies. For instance, some countries reported all WNV infections, others only WNND cases. Some countries reported all confirmed and probable cases, others only the confirmed. The percentage of confirmed cases increased over time, which may suggest improving laboratory capacity/capability in some countries. Some countries also reported cases that were laboratory-confirmed by urine testing, although this is currently not included in the EU case definition. Furthermore, some countries actively monitor and report the final outcome, while others do not, which can compromise the validity of CF estimations and lead to underestimation. The ability to report the outcome of cases only when it is clinically attributable to WNV infections is complex, therefore CF could be overestimated in countries reporting all deaths among patients diagnosed with WNV infection. Only few countries performed continuous monitoring of WNV infections among blood donors and reported asymptomatic infections (Greece reported its first asymptomatic case in 2011, Montenegro in 2013, Austria and Italy in 2015, and Croatia and France in 2018). Furthermore, only the numbers of cases with known clinical manifestations and place of infection were analysed here. Variations in the countries’ reporting completeness limit the validity of direct comparisons.

Improved sensitivity of national surveillance systems, increased diagnostic capacity, more testing following greater awareness of WNV, and the introduction of testing of blood donors could have influenced the yearly number of cases. Furthermore, changes in blood donor screening practice could have influenced the proportion of detected asymptomatic infections, which however was very low in this study.

Asymptomatic cases are usually fortuitous detections through blood donor screenings and do not reflect the true proportion of asymptomatic cases. The proportion of asymptomatic cases detected and reported is small but not negligible. In previously affected or endemic areas in Greece and Italy, positive blood donors have been reported before the diagnosis of symptomatic cases [59]. WNV has also been detected in blood donors originating from areas where the WNV prevalence in humans is low [60]. This highlights the importance of enhanced vigilance and early warning to ensure the timely identification of WNV circulation and affected areas in each transmission season. 

As no specific treatment or vaccine against WNV infection is available for humans, prevention relies to a high degree on personal protective measures against mosquito bites [61]. The effectiveness of different vector control strategies against competent WNV-vector mosquitoes requires further investigation. In addition, a better understanding of the mosquito distribution, host biting preference and species hybridisation will improve knowledge of WNV persistence and the risk to human populations [54]. Comparing whole genome sequences of WNV detected in asymptomatic people (blood donors), patients, animal hosts and vectors may help identify more virulent viral strains and better explain the evolution of the virus and its historical introductions and subsequent spread in Europe.

## Conclusion

Real-time surveillance of WNV infections is key to ensuring that clinicians and public health authorities receive early warning about the occurrence of cases and potential unusual seasonal patterns. This ensures that safety measures are implemented in a timely manner to avoid infections through SoHO.

Understanding the factors that influence WNV ecology and transmission is crucial when trying to predict the risk of increased WNV activity in a season, its geographical distribution and the expected scale of human infections, also at local level. Public health authorities in currently unaffected countries need to be aware that human cases may appear shortly after the first detection of animal cases and should develop preparedness plans before the occurrence of human WNV infections.

## *Note

This designation is without prejudice to positions on status, and is in line with United Nations Security Council Resolution 1244/99 and the International Court of Justice Opinion on the Kosovo declaration of independence.
