# Correction for Euro Surveill. 2020;26(19)

**DOI:** 10.2807/1560-7917.ES.2021.26.20.210520c

**Published:** 2021-05-20

**Authors:** Eurosurveillance editorial team

**Affiliations:** 1 European Centre for Disease Prevention and Control (ECDC), Stockholm, Sweden

On request of the authors, the sentence “The virus is primarily transmitted through the bites of infected mosquitoes, mainly of the *Culex* genus, but occasionally also through transfusion/transplantation of substances of human origin (SoHO) (i.e. blood, organs or cells), percutaneous exposure or inhalation in laboratories, or transplacental passage from mother to fetus” was changed to “The virus is primarily transmitted through the bites of infected mosquitoes, mainly of the *Culex* genus, but occasionally also through transfusion/transplantation of substances of human origin (SoHO) (i.e. blood, organs or cells), percutaneous or conjunctival exposure in laboratories, or transplacental passage from mother to fetus” in the article ‘Epidemiology of human West Nile virus infections in the European Union and European Union enlargement countries, 2010 to 2018’ by Young et al., published on 13 May 2021. This change was made on 20 May 2021. 

